# Supplementary material for: Hepatitis C distribution across diverse population groups in the Eastern Mediterranean Region: An umbrella review
Source: PLoS One. 2026 Apr 21;21(4):e0346782. doi: 10.1371/journal.pone.0346782 (PMC13098937; doi:10.1371/journal.pone.0346782)
Supplement: S1 Table — (DOCX) [file pone.0346782.s001.docx]

**S1 Table:** **Condition/ Context/ Population criteria for inclusion of studies**

| **Parameter** | **Inclusion criteria** |
| --- | --- |
| Condition | Acute/Chronic Hepatitis C |
| Context | Eastern Mediterranean Region countries (Iran, Afghanistan, Bahrain, Djibouti, Egypt, Iraq, Jordan, Kuwait, Libya, Morocco, Oman, Pakistan, Palestine, Qatar, Somalia, Sudan, Syria, United Arab Emirates, Yemen, Tunisia, Saudi Arabia, Lebanon) |
| Population | Key population (people who inject drugs (PWID), people who use drug, people in prison, men who have sex with men (MSM), female sex workers (FSW), and street children, people living with HIV), Co-infected patients(people with , HBV, or other liver-related conditions co-infections), those with clinical or healthcare-associated exposure risk(those undergoing hemodialysis; individuals with thalassemia, hemophilia, or other inherited coagulation disorders; multi-transfused patients; healthcare workers; household contacts of HCV-infected individuals; and patients with comorbidities such as , malignancies, and rheumatologic, dermatologic), and the apparently healthy individuals (general population, blood donors, pregnant women, children, refugees, and army recruits) |
| Study design | Systematic reviews that resulted in a meta-analysis |
