# Supplementary material for: Hepatitis C distribution across diverse population groups in the Eastern Mediterranean Region: An umbrella review
Source: PLoS One. 2026 Apr 21;21(4):e0346782. doi: 10.1371/journal.pone.0346782 (PMC13098937; doi:10.1371/journal.pone.0346782)
Supplement: S2 Table — (DOCX) [file pone.0346782.s002.docx]

**S2 Table.** **Database search strategy using PubMed, Scopus, and Web of Science**

| Database (Search  conducted up to  October 12. 2025) | Search terms |
| --- | --- |
| PubMed | ("hepatitis c" OR HCV OR hepacivirus*) AND (Persian OR Iran OR Iranian OR Irani OR Afghanistan OR Afghan OR Bahrain OR Bahraini OR Djibouti OR Egypt OR Egyptian OR Iraq OR Iraqi OR Jordan OR Jordanian OR Kuwait OR Libya OR Libyan OR Morocco OR Moroccan OR Oman OR Omani OR Pakistani OR Pakistan OR Palestine OR Palestinian OR Qatar OR Qatari OR Somali OR Somalia OR Sudan OR Sudanese OR Syria OR Syrian OR "United Arab Emirates" OR Emirati OR Yemen OR Yemeni OR Tunisia OR Tunisian OR "Saudi Arabia" OR Saudis OR "Saudi Arabians" OR Lebanon OR Lebanese OR EMRO OR "Eastern Mediterranean") AND (review OR "systematic review" OR "meta-analysis" OR "meta-analysis") |
| SCOPUS | (( TITLE-ABS-KEY ( Hepatitis C) OR TITLE-ABS-KEY ( HCV) OR TITLE-ABS-KEY ( hepacivirus) ) ) AND ( ( All field ( Persian ) OR All field ( Iran ) OR All field ( Irani ) OR All field ( Iranian ) OR All field ( Afghanistan ) OR All field ( Afghan ) OR All field ( Bahrain ) OR All field ( Bahraini ) OR All field ( Djibouti ) OR All field ( Egypt ) OR All field ( Egyptian ) OR All field ( Iraq ) OR All field ( Iraqi ) OR All field ( Jordan ) OR All field ( Jordanian ) All field ( Kuwait ) OR All field ( Libya ) OR All field ( Libyan ) OR All field ( Morocco ) OR All field ( Moroccan ) OR All field ( Oman ) OR All field ( Omani ) OR All field ( Pakistani ) OR All field ( Pakistan ) OR All field ( Palestine ) OR All field ( Palestinian ) OR All field ( Qatar ) OR All field ( Qatari ) OR All field ( Somali ) OR All field ( Somalia ) OR All field ( Sudan ) OR All field ( Sudanese ) OR All field ( Syria ) OR All field ( Syrian ) OR All field ( "United Arab Emirates" ) OR All field ( Emirati ) OR All field ( Yemen ) OR All field ( Yemeni ) OR All field ( Tunisia ) OR All field ( Tunisian ) OR All field ( "Saudi Arabia") OR All field ( Saudis ) OR All field ( "Saudi Arabians" ) OR All field ( Lebanon ) OR All field ( Lebanese ) OR All field ( EMRO ) OR All field ( "Eastern Mediterranean" ) ) ) AND ( ( TITLE ( Review ) OR TITLE ( "systematic review" ) OR TITLE( "meta-analysis" ) OR TITLE ( "meta-analysis" ) ) |
| Web of Sciences | ((TS=("hepatitis c" OR HCV OR hepacivirus*)) AND AF=Persian OR Iran OR Iranian OR Irani OR Afghanistan OR Afghan OR Bahrain OR Bahraini OR Djibouti OR Egypt OR Egyptian OR Iraq OR Iraqi OR Jordan OR Jordanian OR Kuwait OR Libya OR Libyan OR Morocco OR Moroccan OR Oman OR Omani OR Pakistani OR Pakistan OR Palestine OR Palestinian OR Qatar OR Qatari OR Somali OR Somalia OR Sudan OR Sudanese OR Syria OR Syrian OR "United Arab Emirates" OR Emirati OR Yemen OR Yemeni OR Tunisia OR Tunisian OR “Saudi Arabia” OR Saudis OR “Saudi Arabians” OR Lebanon OR Lebanese OR EMRO OR "Eastern Mediterranean" AND TS, ABS= review OR “systematic review” OR “meta-analysis” OR “meta-analysis”)) |
