# Supplementary material for: Hepatitis C distribution across diverse population groups in the Eastern Mediterranean Region: An umbrella review
Source: PLoS One. 2026 Apr 21;21(4):e0346782. doi: 10.1371/journal.pone.0346782 (PMC13098937; doi:10.1371/journal.pone.0346782)
Supplement: S3 Table — (DOCX) [file pone.0346782.s003.docx]

**S3 Table.** **Summary of reviews included in the Umbrella Review of HCV prevalence across diverse populations in EMR**

| **Apparently healthy individuals (**general population, blood donors, pregnant women, children, refugees, and army recruits)**, (53 estimates)** | | | | | | | |  |
| --- | --- | --- | --- | --- | --- | --- | --- | --- |
| **First Author, Year** | **Country** | **Number of studies included** | **Number of events** | **Sample size** | **HCV Prevalence** **[95% CI]** | **Study group** | **Quality level^[[1]](#footnote-1)^** | **Heterogeneity**  **(I^2^)** |
| Abbasi, 2023 (1) | Afghanistan | 1 | 13 | 4452 | 0.00 [0.00, 0.01] | Pregnant women | High | - |
| Chemaitelly, 2015 (2) | Afghanistan | 47 | 5,246 | 749,466 | 0.01 [0.00, 0.01] | General population | High | >75% |
| Mohamoud, 2016 (3) | Bahrain | 2 | 63 | 21,125 | 0.00 [0.00, 0.00] | General population | High | 98.1% |
| Chaabna, 2016 (4) | Djibouti | 1 | 24 | 8057 | 0.00 [0.00, 0.01] | General population | High | - |
| Ahmed, 2025 (5) | Egypt | 7 | 30 | 576 | 0.05 [0.03, 0.07] | Blood doners | High | - |
| Muhammad, 2022 (6) | Egypt | 5 | 72 | 2,799 | 0.03 [0.02, 0.03] | Children | High | 93.64% |
| Ghaderi, 2017 (7) | Egypt | 3 | 31,333 | 543,978 | 0.06 [0.03, 0.08] | Blood donors | High | - |
| Kouyoumjian, 2017 (8) | Egypt | 264 | 199,611 | 1,677,404 | 0.12 [0.11, 0.13] | General population | High | 99.3% (99.3–99.3%) |
| Obeid, 2024 (9) | GCC and Yemen | 1 | 320 | 16,367 | 0.02 [0.02, 0.02] | Screening population | High | 100.0% |
| Obeid, 2024 (9) | Iran | 3 | 0 | 695,067 | 0.00 [0.00, 0.17] | Screening population | High | 100.0% |
| Kasraian, 2021 (10) | Iran | 58 | 25,373 | 12,686,297 | 0.00 [0.00, 0.00] | Blood doners | High | 98.64% |
| Ghaderi, 2017 (7) | Iran | 13 | 13,329 | 9,520,630 | 0.00 [0.00, 0.00] | Blood donors | High | - |
| Mirminachi, 2017 (11) | Iran | 12 | 514 | 85,611 | 0.01 [0.00, 0.01] | General population | High | 80.2% |
| Bagheri Amiri, 2016 (12) | Iran | 13 | 52,817 | 170,378 | 0.31 [0.18, 0.43] | General population | Moderate | - |
| Khodabandeh et al, 2013 (13) | Iran | 48 | 42036 | 10739221 | 0.01 [0.00, 0.01] | Blood doners | High | 99.9% |
| Abbasi, 2023 (1) | Iraq | 2 | 130 | 3645 | 0.03 [0.03, 0.04] | Pregnant women | High | - |
| Chemaitelly, 2015 (2) | Iraq | 99 | 3,719 | 1,859,563 | 0.00 [0.00, 0.00] | General population | High | 95.8% (95.3–96.3%) |
| Chemaitelly, 2015 (2) | Jordan | 12 | 378 | 126,152 | 0.00 [0.00, 0.01] | General population | High | 93.6% (90.5–95.6%) |
| Mohamoud, 2016 (3) | Kuwait | 5 | 649 | 44,772 | 0.01 [0.01, 0.02] | General population | High | 97.6%  (97.1-98.1) |
| Mohamoud, 2016 (3) | Kuwait | 2 | 56 | 12,853 | 0.00 [0.01, 0.02] | General population | High | 39.9%  (0.0-72.3) |
| Ghaderi, 2017 (7) | Lebanon | 1 | 64 | 16,084 | 0.00 [0.00, 0.01] | Blood donors | High | - |
| Chemaitelly, 2015 (2) | Lebanon | 16 | 7,612 | 38,059 | 0.00 [0.00, 0.00] | General population | High | 60.8% (32.3–77.3%) |
| Ghaderi, 2017 (7) | Libya | 2 | 15,746 | 1,009,414 | 0.02 [0.01, 0.02] | Blood donors | High | - |
| Fadlalla, 2015 (14) | Morocco | 11 | 3,009 | 417,897 | 0.01 [0.00, 0.01] | General population | High | 99.3% |
| Ghaderi, 2017 (7) | Occupied Palestinian territory | 2 | 670 | 419,258 | 0.00 [0.00, 0.02] | Blood donors | High | - |
| Ghaderi, 2017 (7) | Oman | 1 | 158 | 21,131 | 0.01 [0.01, 0.01] | Blood donors | High | - |
| Mohamoud, 2016 (3) | Oman | 6 | 258 | 64,530 | 0.00 [0.00, 0.00] | General population | High | 23.4%  (0.0-67.4) |
| Abbasi, 2023 (1) | Pakistan | 21 | 7,585 | 108,361 | 0.07 [0.06, 0.12] | Pregnant women | High | 99.35% |
| Sarwat, 2019 (15) | Pakistan | 48 | 128,065 | 2,099,434 | 0.06 [0.05, 0.07] | General population | High | 99.6%(96.6–96.6%) |
| Al Kanaani, 2018 (16) | Pakistan | 148 | 71,660 | 1,352,080 | 0.05 [0.00, 0.19] | General population | High | 99.2%(99.1–99.2%) |
| Al Kanaani, 2017 (16) | Pakistan | 122 | 48,220 | 16,073,479 | 0.00 [0.00, 0.00] | General population | High | 99.2%(99.1–99.2%) |
| Chemaitelly, 2015 (2) | Palestine | 53 | 675 | 337,384 | 0.00 [0.00, 0.00] | General population | High | 91.8% (90.1–93.3%) |
| Mohamoud, 2016 (3) | Qatar | 4 | 1,629 | 153,704 | 0.01 [0.00, 0.02] | General population | High | 99.3%  (99.2-99.4) |
| Mohamoud, 2016 (3) | Qatar | 2 | 151 | 29,764 | 0.01 [0.00, 0.06] | General population | High | 0 |
| Ghaderi, 2017 (7) | Saudi Arabia | 8 | 4,113 | 663,428 | 0.01 [0.0, 0.01] | Blood donors | High | - |
| Mohamoud, 2016 (3) | Saudi Arabia | 27 | 13,345 | 808,787 | 0.02 [0.01, 0.02] | General population | High | 98.4%  (98.2-98.5) |
| Mohamoud, 2016 (3) | Saudi Arabia | 51 | 16,286 | 999,127 | 0.02 [0.01, 0.02] | General population | High | 97.8%  (97.6-97.9) |
| Sharif Ali Mude, 2025 (17) | Somalia | 15 | 1,565 | 88,782 | 0.04 [0.02, 0.06] | General population | High | 98.4% |
| Hassan-Kadle, 2018 (18) | Somalia | 29 | 303 | 6,257 | 0.05 [0.03, 0.07] | General population | High | 93.5%, (90.4- 95.6) |
| Chaabna, 2016 (4) | Somalia | 9 | 127 | 14,081 | 0.01 [0.00, 0.02] | General population | High | 67% (32%-83%) |
| Abbasi, 2023 (1) | Sudan | 1 | 3 | 423 | 0.01 [0.0, 0.02] | Pregnant women | High | - |
| Chaabna, 2016 (4) | Sudan | 7 | 19 | 1,856 | 0.01 [0.00, 0.02] | General population | High | 53% (0%-80%) |
| Ghaderi, 2017 (7) | Syria | 2 | 26 | 4039 | 0.01 [0.00, 0.01] | Blood donors | High | - |
| Chemaitelly, 2015 (2) | Syria | 17 | 4,458 | 1,114,550 | 0.00 [0.00, 0.01] | General population | High | 92.6% (89.7–94.7%) |
| Fadlalla, 2015 (14) | Tunisia | 24 | 3,800 | 760,041 | 0.01 [0.00, 0.01] | General population | High | 98.6% |
| Mohamoud, 2016 (3) | UAE | 10 | 4,769 | 290,778 | 0.02 [0.01, 0.03] | General population | High | 99.4%  (99.3-99.5) |
| Mohamoud, 2016 (3) | UAE | 2 | 3 | 1432 | 0.00 [0.00, 0.01] | General population | High | 0 |
| Abbasi, 2023 (1) | Yemen | 2 | 68 | 800 | 0.09 [0.07, 0.10] | Pregnant women | High | - |
| Chaabna, 2016 (4) | Yemen | 24 | 919 | 48,343 | 0.02 [0.01, 0.03] | General population | High | 93% (91%-96%) |
| **People with clinical or healthcare-associated exposure risk (those undergoing hemodialysis; individuals with thalassemia, hemophilia, or other inherited coagulation disorders; multi-transfused patients; healthcare workers; household contacts of HCV-infected individuals; and patients with comorbidities). (79 estimates)** | | | | | | | | |
| Chemaitelly, 2015 (2) | Afghanistan | 16 | 146 | 6,356 | 0.02 [0.01, 0.04] | Clinical population | High | >75% |
| Ahmed, 2025 (5) | Egypt | 11 | 84 | 889 | 0.10 [0.06, 0.14] | Hemodialysis patients | High | 73.7% |
| Ahmed, 2025 (5) | Egypt | 5 | 107 | 334 | 0.32 [0.12, 0.62] | Patients with blood-related malignancies | High | 91.4% |
| Hedayati-Moghaddam, 2021 (19) | Egypt | 8 | 57 | 499 | 0.11 [0.07, 0.19] | Hemodialysis patients | High | 81.2% |
| Harfouche, 2018 (20) | Egypt | 26 | 3,219 | 4,915 | 0.66 [0.57, 0.74] | Hemodialysis patient | High | 96·9% (96·2–97·5) |
| Ashkani-Esfahani, 2017 (21) | Egypt | 7 | 13,504 | 27,007 | 0.50 [0.46, 0.55] | Hemodialysis patients | High | 94.7% |
| Kouyoumjian, 2017 (8) | Egypt | 45 | 1,348 | 9,427 | 0.14 [0.10, 0.19] | Clinical population | High | 96.9% (96.4–97.4%) |
| Kouyoumjian, 2017 (8) | Egypt | 34 | 1,940 | 5,542 | 0.35 [0.27, 0.43] | Clinical population | High | 96.9% (96.4–97.4%) |
| Alavian, 2011 (22) | Egypt | 5 | 297 | 619 | 0.48 [0.45, 0.51] | Hemodialysis patients | Moderate | - |
| Alavian, 2010 (23) | Egypt | 2 | 43 | 63 | 0.69 [0.58, 0.80] | Thalassemia Patients | Moderate | 0-100% |
| Obeid, 2024 (9) | GCC^[[2]](#footnote-2)^ & Yemen | 3 | 92 | 767 | 0.12 [0.01, 0.72] | Clinical population | High | 97.0% |
| Obeid, 2024 (9) | Iran | 8 | 322 | 2,480 | 0.13 [0.04, 0.38] | Clinical population | High | 99.0% |
| Hedayati-Moghaddam, 2021 (19) | Iran | 7 | 92 | 1,550 | 0.06 [0.03, 0.11] | Hemodialysis patients | High | 81.2% |
| Molaei, 2021 (24) | Iran | 151 | 2,770 | 23,876 | 0.12 [0.10, 0.13] | Hemodialysis patients | High | 90.3% |
| Molaei, 2021 (24) | Iran | 151 | 10,782 | 23,876 | 0.45 [0.38, 0.54] | Hemophiliac patients | High | 90.5% |
| Molaei, 2021 (24) | Iran | 151 | 4,362 | 23,876 | 0.18 [0.16, 0.21] | Thalassemia patients | High | 89.0% |
| Shamshirian, 2019 (25) | Iran | 37 | 1,561 | 9,185 | 0.17 [0.15, 0.20] | Thalassemia patients | High | 89.8% |
| Harfouche, 2018 (20) | Iran | 41 | 1,393 | 15,140 | 0.09 [0.06, 0.11] | Hemodialysis patient | High | 96·3% (95·6–96·9) |
| Behzadifar, 2018(26) | Iran | 52 | 2,525 | 13,291 | 0.19 [0.16, 0.21] | Thalassemia patients | High | 94.1% |
| Ashkani-Esfahani, 2017 (21) | Iran | 21 | 751 | 6,255 | 0.12 [0.10, 0.15] | Hemodialysis patients | High | 90.0% |
| Ramezan-Ghorbani, 2019 (27) | Iran | 54 | 2,631 | 23,921 | 0.11 [0.10, 0.13] | Hemodialysis peoples | High | 100.0% |
| Bagheri Amiri, 2016 (12) | Iran | 4 | 113 | 595 | 0.19 [0.00, 0.66] | Health care worker | Moderate | - |
| Bagheri Amiri, 2016 (12) | Iran | 19 | 756 | 3,919 | 0.19 [0.14, 0.25] | Patients who received multiple transfusions | Moderate | - |
| LIU, 2014 (28) | Iran | 5 | 2 | 1,801 | 0.00 [0.00, 0.00] | Hemodialysis patients | High | 81.8% |
| Alavian, 2012 (29) | Iran | 21 | 1,524 | 3,171 | 0.48 [0.36, 0.60] | Inherited Coagulation Disorders | High | 98.5% |
| Alavian, 2011 (22) | Iran | 22 | 990 | 5,821 | 0.17 [0.13, 0.20] | Hemodialysis patients | Moderate | - |
| Alavian, 2010 (30) | Iran | 12 | 681 | 5,280 | 0.13 [0.10, 0.16] | Hemodialysis patients | Moderate | High |
| Alavian, 2010 (23) | Iran | 21 | 941 | 5,229 | 0.18 [0.14, 0.21] | Thalassemia Patients | Moderate | 0-100% |
| Harfouche, 2018 (20) | Iraq | 16 | 225 | 1,353 | 0.17 [0.09, 0.26] | Hemodialysis patient | High | 94·0% (91·6–95·6) |
| Ashkani-Esfahani, 2017 (31) | Iraq | 9 | 187 | 937 | 0.20 [0.12, 0.28] | Hemodialysis patients | High | 97.3% |
| Chemaitelly, 2015 (2) | Iraq | 28 | 126 | 8,398 | 0.02 [0.01, 0.03] | Clinical population | High | 86.8% (82.1–90.3%) |
| Chemaitelly, 2015 (2) | Iraq | 45 | 942 | 18,845 | 0.05 [0.03, 0.07] | Clinical population | High | 96.2% (95.5–96.7%) |
| Alavian, 2012 (29) | Iraq | 2 | 152 | 290 | 0.52 [0.27, 0.80] | Inherited Coagulation Disorders | High | 91.3% |
| Harfouche, 2018 (20) | Jordan | 9 | 986 | 2,730 | 0.36 [0.27, 0.45] | Hemodialysis patient | High | 93·4% (89·5–95·8) |
| Ashkani-Esfahani, 2017 (31) | Jordan | 4 | 606 | 1,731 | 0.35 [0.17, 0.54] | Hemodialysis patients | High | 97.4% |
| LIU, 2014 (28) | Jordan | 3 | 196 | 676 | 0.29 [0.230, 0.360] | Hemodialysis patients | High | 71.1% |
| Harfouche, 2018 (20) | Kuwait | 3 | 238 | 1,597 | 0.15 [0.03, 0.34] | Hemodialysis patient | High | 98·7% (97·8–99·2) |
| Harfouche, 2018 (20) | Lebanon | 9 | 308 | 4,214 | 0.07 [0.04, 0.12] | Hemodialysis patient | High | 95·0% (92·3–96·7) |
| Ashkani-Esfahani, 2017 (31) | Lebanon | 2 | 431 | 4799 | 0.09 [0.01, 0.20] | Hemodialysis patients | High | 98.4% |
| Chemaitelly, 2015 (2) | Lebanon | 7 | 13 | 1,050 | 0.01 [0.00, 0.03] | Clinical population | High | 76.0% (49.3–88.6%) |
| Harfouche, 2018 (20) | Libya | 5 | 801 | 3,559 | 0.22 [0.14, 0.32] | Hemodialysis patient | High | 95·8% (92·7–97·6) |
| Harfouche, 2018 (20) | Morocco | 7 | 644 | 1,387 | 0.46 [0.28, 0.65] | Hemodialysis patient | High | 97·5% (96·3–98·3) |
| Alavian, 2011 (22) | Morocco | 2 | 352 | 489 | 0.72 [0.68, 0.76] | Hemodialysis patients | Moderate | - |
| Harfouche, 2018 (20) | Oman | 1 | 27 | 102 | 0.26 [0.18, 0.36] | Hemodialysis patient | High | 0 |
| Waheed, 2021 (32) | Pakistan | 33 | 2,548 | 8,554 | 0.30 [0.05, 0.65] | Thalassemia patient | High | - |
| Akhtar, 2020 (33) | Pakistan | 27 | 2,096 | 5,789 | 0.36 [0.29, 0.44] | Thalassemia patient | High | 97% |
| Akhtar, 2020 (34) | Pakistan | 19 | 1,114 | 3,446 | 0.32 [0.26, 0.39] | Hemodialysis patients | High | 94.3% |
| Al Kanaani, 2018 (16) | Pakistan | 21 | 841 | 2,377 | 0.35 [0.08, 0.68] | Clinical population | High | 93.2% (90.9–94.9%) |
| Al Kanaani, 2018 (16) | Pakistan | 64 | 20,204 | 156,623 | 0.13 [0.00, 0.71] | Clinical population | High | 99.3% (99.2–99.3%) |
| Harfouche, 2018 (20) | Pakistan | 7 | 302 | 995 | 0.30 [0.22, 0.399] | Hemodialysis patient | High | 87·8% (77·3–93·5) |
| Al Kanaani,, 2018 (16) | Pakistan | 70 | 2,286 | 36,879 | 0.06 [0.03, 0.09] | Clinical population | High | - |
| Al Kanaani,, 2018 (16) | Pakistan | 72 | 2,539 | 55,187 | 0.05 [0.03, 0.06] | Clinical populations | High | - |
| Alavian, 2012 (29) | Pakistan | 4 | 303 | 841 | 0.36 [0.00, 0.67] | Inherited Coagulation Disorders | High | 99.2% |
| Alavian, 2011 (22) | Pakistan | 2 | 54 | 147 | 0.37 [0.30, 0.44] | Hemodialysis patients | Moderate | - |
| Alavian, 2010 (30) | Pakistan | 8 | 633 | 1,406 | 0.45 [0.43, 0.48] | Thalassemia Patients | Moderate | High |
| Harfouche, 2018 (20) | Palestine | 12 | 130 | 1,260 | 0.10 [0.06, 0.16] | Hemodialysis patient | High | 88·3% (81·4–92·6) |
| Ashkani-Esfahani, 2017 (31) | Palestine | 3 | 232 | 1,291 | 0.18 [0.05, 0.30] | Hemodialysis patients | High | 94.8% |
| Harfouche, 2018 (20) | Qatar | 1 | 58 | 130 | 0.45 [0.36, 0.54] | Hemodialysis patient | High | 0 |
| Hedayati-Moghaddam, 2021 (19) | Saudi Arabia | 1 | 12 | 84 | 0.14 [0.08, 0.23] | Hemodialysis patient | High | 81.2% |
| Harfouche, 2018 (20) | Saudi Arabia | 39 | 20,501 | 43,250 | 0.47 [0.44, 0.51] | Hemodialysis patient | High | 96·2% (95·5–96·8) |
| Ashkani-Esfahani, 2017 (31) | Saudi Arabia | 1 | 34 | 180 | 0.19 [0.13, 0.25] | Hemodialysis patients | High | 0 |
| LIU, 2014 (28) | Saudi Arabia | 8 | 635 | 1,222 | 0.52 [0.40, 0.64] | Hemodialysis patients | High | 94.8% |
| Alavian, 2012 (29) | Saudi Arabia | 1 | 22 | 28 | 0.79 [0.63, 0.94] | Inherited Coagulation Disorders | High | - |
| Alavian, 2011 (22) | Saudi Arabia | 12 | 2,102 | 3,337 | 0.63 [0.61, 0.64] | Hemodialysis patients | Moderate | - |
| Alavian, 2010 (30) | Saudi Arabia | 3 | 87 | 138 | 0.63 [0.56, 0.69] | Thalassemia Patients | Moderate | High |
| Chaabna, 2016 (4) | Somalia | 5 | 12 | 702 | 0.02 [0.00, 0.05] | Clinical population | High | 77% (44%-90%) |
| Harfouche, 2018 (20) | Sudan | 3 | 130 | 635 | 0.20 [0.08, 0.37] | Hemodialysis patient | High | 94·5% (87·3–97·6) |
| Chaabna, 2016 (4) | Sudan | 23 | 39 | 6,450 | 0.01 [0.00, 0.01] | Clinical population | High | 79% (69%-86%) |
| Harfouche, 2018 (20) | Syria | 5 | 458 | 809 | 0.57 [0.47, 0.65] | Hemodialysis patient | High | 83·5% (62·6–92·7) |
| Ashkani-Esfahani, 2017 (31) | Syria | 1 | 297 | 550 | 0.54 [0.50, 0.59] | Hemodialysis patients | High | 0 |
| Alavian, 2011 (22) | Syria | 3 | 493 | 809 | 0.61 [0.57, 0.64] | Hemodialysis patients | Moderate | - |
| Harfouche, 2018 (20) | Tunisia | 14 | 1,535 | 5,602 | 0.27 [0.27, 0.32] | Hemodialysis patient | High | 93·3% (90·4–95·3) |
| Alavian, 2012 (29) | Tunisia | 2 | 91 | 165 | 0.58 [0.48, 0.63] | Inherited Coagulation Disorders | High | 0 |
| Alavian, 2011 (22) | Tunisia | 4 | 1,156 | 5,024 | 0.23 [0.21, 0.24] | Hemodialysis patients | Moderate | - |
| Harfouche, 2018 (20) | United Arab Emirate | 1 | 64 | 262 | 0.24 [0.19, 0.30] | Hemodialysis patient | High | 0 |
| Harfouche, 2018 (20) | Yemen | 3 | 142 | 300 | 0.47 [0.33, 0.62] | Hemodialysis patient | High | 76·8% (24·2–92·9) |
| Ashkani-Esfahani, 2017 (31) | Yemen | 4 | 287 | 683 | 0.42 [0.28, 0.56] | Hemodialysis patients | High | 93.4% |
| Chaabna, 2016 (4) | Yemen | 13 | 549 | 2,832 | 0.19 [0.13, 0.27] | Clinical population | High | 95%(93%-96%) |
| **Co-infected patients** (people living with HIV, HBV, or with HIV/HBV/HCV co-infections). **(9 estimates)** | | | | | | | | |
| Ahmed, 2025 (5) | Egypt | 7 | 65 | 297 | 0.22 [0.13, 0.34] | Patients with other liver disease | High | 77.2% |
| Sarwat, 2020 (35) | Egypt | 53 | 22,362 | 38,030 | 0.60 [0.51, 0.66] | Populations With Liver-Related Diseases | Moderate | 99.2% (99.2‐99.3) |
| Kouyoumjian, 2017 (8) | Egypt | 72 | 26,440 | 47,214 | 0.56 [0.50, 0.62] | Populations with liver-related conditions | High | 99.1% (99.0–99.2%) |
| Hashiani, 2019 (36) | Iran | 10 | 35 | 2,724 | 0.01 [0.00, 0.02] | HBV patients | High | 50.0% |
| Hashiani, 2019 (36) | Iran | 7 | 13 | 2,571 | 0.00 [0.00, 0.01] | HBV and HIV patients | High | 46.0% |
| Hashiani, 2019 (36) | Iran | 14 | 80 | 6,218 | 0.01 [0.00, 0.02] | HBV patients | High | - |
| Sarwat, 2020 (35) | Pakistan | 76 | 13,311 | 23,854 | 0.56 [0.49, 0.62] | Populations With Liver-Related Diseases | Moderate | 99.0% (98.9‐99.1) |
| Al Kanaani, 2018 (16) | Pakistan | 20 | 1,851 | 11,940 | 0.15 [0.01, 0.55] | Clinical liver disease | High | 99.7%(99.6–99.7% |
| Al Kanaani, 2018 (16) | Pakistan | 73 | 14,689 | 23,132 | 0.64 [0.49, 0.78] | Populations with liver-related conditions | High | 99.0%(98.9–99.1%) |
| **Key population (people who inject drugs (PWID), non-injecting drug users, people who use drugs non-injecting, People in prisons, men who have sex with men (MSM), female sex workers (FSW), people living with HIV (PLWH), and street children).**  **(75 estimates)** | | | | | | | | |
| Adeiza, 2024 (37) | Afghanistan | 3 | 69,520 | 763,961 | 0.09 [0.00, 0.68] | Population at high risk | High | 99.9% |
| Aghae, 2023 (38) | Afghanistan | 4 | 13,203 | 57,207 | 0.23 [0.09, 0.41] | PWID | High | 99.6% |
| Shayan, 2021 (39) | Afghanistan | 4 | 790 | 2,118 | 0.37 [0.35, 0.39] | PWID | High | - |
| Sarwat, 2020 (40) | Afghanistan | 17 | 2,828 | 8,597 | 0.33 [0.25, 0.41] | People who use drugs | Moderate | 97.8% (97.2–98.2%) |
| Chemaitelly, 2015 (2) | Afghanistan | 14 | 2,653 | 8,139 | 0.33 [0.24, 0.41] | PWID | High | >75% |
| Aghae, 2023 (38) | Bahrain | - | 1921 | 5100 | 0.38 [0.33, 0.43] | PWID | High | - |
| Aghae, 2023 (38) | Egypt | 3 | 55,813 | 96,230 | 0.58 [0.52, 0.64] | PWID | High | - |
| Sarwat, 2020 (40) | Egypt | 2 | 125 | 243 | 0.52 [0.30, 0.73] | People who use drugs | Moderate | - |
| Kouyoumjian, 2017 (41) | Egypt | 57 | 4,147 | 7,459 | 0.56 [0.49, 0.62] | High-risk population | High | 96.1% (95.5–96.6%) |
| Adeiza , 2024 (37) | Gulf Co-operation Council^[[3]](#footnote-3)^ | 4 | 383,162 | 4,076,192 | 0.09 [0.02, 0.37] | Population at high risk | High | 99.9% |
| Mostafavi, 2024 (42) | Iran | 42 | 5,372 | 17,905 | 0.30 [0.45, 0.64] | HIV patients | High | 99.4% |
| Hajizadeh, 2024 (43) | Iran | 66 | 9,360 | 15,344 | 0.61 [0.55, 0.67] | HIV patients | High | - |
| Adeiza, 2024 (37) | Iran | 2 | 4,837,310 | 16,092,172 | 0.29 [0.27, 0.31] | Population at high risk | High | 97.2% |
| Aghae, 2023 (38) | Iran | 40 | 50,876 | 138,250 | 0.37 [0.31, 0.43] | PWID | High | 96.5% |
| Nasiri, 2023 (44) | Iran | 5 | 128 | 6,782 | 0.02 [0.01, 0.03] | Street children | High | 65.4% |
| Mehmandoost, 2022(45) | Iran | 10 | 4087 | 19011 | 0.21 [0.13, 0.30] | People in prison | High | 96.7% |
| Rajabi, 2021 (46) | Iran | 62 | 12,570 | 27,033 | 0.46 [0.41, 0.52] | PWID | High | 99.0% |
| Shayan, 2021 (39) | Iran | 42 | 8,378 | 17,563 | 0.48 [0.43, 0.52] | PWID | High | - |
| Karamouzian, 2020 (47) | Iran | 3 | 25 | 401 | 0.06 [0.01, 0.11] | FSW | High | - |
| Najimi, 2020 (48) | Iran | 33 | 9,333 | 37,512 | 0.25 [0.19, 0.32] | People in prison | High | - |
| Najimi, 2020 (49) | Iran | 62 | 9,576 | 22,795 | 0.42 [0.37, 0.47] | People who use drugs | High | 98.8% |
| Hashiani, 2019 (36) | Iran | 14 | 1013 | 6,218 | 0.16 [0.01, 0.32] | HIV patients | High | 99.8% |
| Sarwat, 2019 (40) | Iran | 60 | 10,316 | 19,614 | 0.53 [0.48, 0.57] | People who use drugs | Moderate | 97.9% (97.6–98.1%) |
| Behzadifar, 2018 (50) | Iran | 4 | 41 | 1,691 | 0.02 [0.01, 0.03] | Street children | High | 99.3% |
| Behzadifar, 2018 (51) | Iran | 17 | 5,234 | 18,693 | 0.28 [0.21, 0.36] | People in prison | High | 99.3% |
| Mohammadi, 2018(52) | Iran | 11 | 1,675 | 9004 | 0.18 [0.12, 0.27] | People in prison | Moderate | 98.0% |
| Nematollahi, 2018 (53) | Iran | 46 | 11,226 | 34,755 | 0.32 [0.31, 0.34] | High-risk population | Moderate | 99.5% |
| Nematollahi, 2018 (53) | Iran | 26 | 6,992 | 16,929 | 0.41 [0.30, 0.53] | PWID | Moderate | 99.5% |
| Nematollahi, 2018 (53) | Iran | 3 | 220 | 894 | 0.25 [0.16, 0.33] | People who use drugs in prison | Moderate | 99.5% |
| Nematollahi, 2018 (53) | Iran | 10 | 3,329 | 14,535 | 0.23 [0.13, 0.33] | People in prison | Moderate | 99.5% |
| Nematollahi, 2018 (53) | Iran | 4 | 336 | 2,075 | 0.16 [0.13, 0.20] | People who use drugs | Moderate | 99.5% |
| Nematollahi, 2018 (53) | Iran | 1 | 13 | 161 | 0.08 [0.04, 0.13] | FSW | Moderate | - |
| Bagheri Amiri, 2016 (12) | Iran | 5 | 722 | 1,403 | 0.51 [0.34, 0.68] | PWID | Moderate | - |
| Bagheri Amiri, 2016 (12) | Iran | 2 | 4 | 488 | 0.01 [0.00, 0.02] | Street children | Moderate | - |
| Bagheri Amiri, 2016 (12) | Iran | 1 | 496 | 1431 | 0.35 [0.32, 0.37] | People in prison | Moderate | - |
| Malekinejad, 2015 (54) | Iran | 23 | 34 | 7,565 | 0.00 [0.00, 0.01] | PWID | High | 98.5% |
| Malekinejad, 2015 (54) | Iran | 5 | 1 | 872 | 0.00 [0.00, 0.00] | People who use drugs | High | 97.8% |
| Aghae, 2023 (38) | Iraq | - | 7406 | 39,277 | 0.20 [0.14, 0.24] | PWID | High | - |
| Chemaitelly, 2015 (2) | Iraq | 58 | 1,308 | 6,707 | 0.19 [0.15, 0.25] | PWID | High | 95.8% (95.1–96.4%) |
| Aghae, 2023 (38) | Jordan | 1 | 1976 | 10,488 | 0.20 [0.14, 0.25] | PWID | High | - |
| Chemaitelly, 2015 (2) | Jordan | 12 | 1,069 | 2,888 | 0.37 [0.29, 0.45] | PWID | High | 92.0% (87.9–94.7%) |
| Aghae, 2023 (38) | Kuwait | 1 | 3692 | 12,000 | 0.31 [0.01, 0.75] | PWID | High | - |
| Aghae, 2023 (38) | Lebanon | 3 | 2,127 | 9,000 | 0.23 [0.18, 0.30] | PWID | High | 83.5% |
| Fengyi, 2020 (55) | Lebanon | 1 | 0 | 101 | 0.00 [0.00, 0.00] | MSM | High | - |
| Sarwat, 2019 (40) | Lebanon | 3 | 60 | 240 | 0.25 [0.04, 0.54] | People who use drugs | Moderate | 95.3% (89.6–97.9%) |
| Chemaitelly, 2015 (2) | Lebanon | 10 | 190 | 1,309 | 0.14 [0.06, 0.26] | PWID | High | 96.0% (94.2–97.2%) |
| Aghae, 2023 (38) | Libya | 1 | 6290 | 6677 | 0.94 [0.91, 0.96] | PWID | High | - |
| Fengyi, 2020 (55) | Libya | 1 | 19 | 224 | 0.08 [0.05, 0.13] | MSM | High | - |
| Sarwat, 2019 (40) | Libya | 1 | 309 | 328 | 0.94 [0.91, 0.96] | People who use drugs | Moderate | - |
| Aghae, 2023 (38) | Morocco | 7 | 11,200 | 17,750 | 0.63 [0.51, 0.75] | PWID | High | 95.4% |
| Sarwat, 2019 (40) | Morocco | 3 | 402 | 759 | 0.53 [0.33, 0.72] | People who use drugs | Moderate | 96.9% (93.6–98.5%) |
| Aghae, 2023 (38) | Oman | 0 | 1117 | 2922 | 0.36 [0.21, 0.53] | PWID | High | 98.7% |
| Sarwat, 2019 (40) | Oman | 1 | 246 | 512 | 0.48 [0.44, 0.52] | People who use drugs | Moderate | - |
| Aghae, 2023 (38) | Pakistan | 21 | 161,981 | 430,000 | 0.38 [0.28, 0.74] | PWID | High | 96.1% |
| Shayan, 2021 (39) | Pakistan | 13 | 1,634 | 3,004 | 0.54 [0.33, 0.73] | PWID | High | - |
| Sarwat, 2019 (40) | Pakistan | 19 | 1,857 | 3,304 | 0.56 [0.41, 0.70] | People who use drugs | Moderate | 98.6% (98.3–98.8%) |
| Al Kanaani, 2018 (16) | Pakistan | 15 | 1,222 | 2,733 | 0.45 [0.08, 0.94] | PWID | High | 98.8%(98.6–99.0%) |
| Al Kanaani, 2018 (16) | Pakistan | 208 | 17,737 | 55,257 | 0.32 [0.28, 0.36] | High-risk population | High | - |
| Aghae, 2023 (38) | Palestine | 3 | 2,075 | 5,000 | 0.41 [0.37, 0.46] | PWID | High | 0 |
| Sarwat, 2019 (40) | Palestine | 4 | 270 | 480 | 0.56 [0.41, 0.70] | People who use drugs | Moderate | 29.8% (0.0–74.4%) |
| Aghae, 2023 (38) | Qatar | N/A | 688 | 1,827 | 0.38 [0.33, 0.43] | PWID | High | - |
| Aghae, 2023 (38) | Saudi Arabia | 2 | 2083 | 3400 | 0.63 [0.30, 0.89] | PWID | High | 98.7% |
| Sarwat, 2019 (40) | Saudi Arabia | 5 | 6,825 | 12,298 | 0.55 [0.20, 0.87] | People who use drugs | Moderate | 99.9% (99.9–99.9%) |
| Adeiza, 2024 (37) | Somalia | 1 | 6,339 | 17,230 | 0.48 [0.47, 0.50] | Population at high risk | High | 0 |
| Aghae, 2023 (38) | Somalia | - | 74 | 392 | 0.20 [0.14, 0.25] | PWID | High | - |
| Adeiza, 2024 (37) | Sudan | 1 | 173 | 8,643 | 0.02 [0.01, 0.02] | Population at high risk | High | 0 |
| Aghae, 2023 (38) | Sudan | - | 191 | 986 | 0.20 [0.14, 0.25] | PWID | High | - |
| Chaabna, 2016 (4) | Sudan | 6 | 140 | 979 | 0.14 [0.06, 0.25] | PWID | High | 93.3%(88%96%) |
| Aghae, 2023 (38) | Syria | 1 | 328 | 10,000 | 0.03 [0.17, 0.05] | PWID | High | - |
| Sarwat, 2019 (40) | Syria | 2 | 37 | 95 | 0.40 [0.07, 0.78] | People who use drugs | Moderate | - |
| Chemaitelly, 2015 (2) | Syria | 8 | 606 | 1,279 | 0.47 [0.32, 0.62] | PWID | High | 96.3% (94.5–97.6%) |
| Aghae, 2023 (38) | Tunisia | 6 | 3,115 | 11,000 | 0.28 [0.25, 0.31] | PWID | High | 0 |
| Sarwat, 2019 (40) | Tunisia | 1 | 4 | 23 | 0.22 [0.05, 0.39] | People who use drugs | Moderate | - |
| Aghae, 2023 (38) | United Arab Emirate | - | 2353 | 6247 | 0.38 [0.33, 0.43] | PWID | High | - |
| Aghae, 2023 (38) | Yemen | - | 160 | 844 | 0.20 [0.14, 0.25] | PWID | High | - |

1. Abbasi F, Almukhtar M, Fazlollahpour-Naghibi A, Alizadeh F, Moghadam KB, Tadi MJ, et al. Hepatitis C infection seroprevalence in pregnant women worldwide: a systematic review and meta-analysis. EClinicalMedicine. 2023;66.

2. Chemaitelly H, Chaabna K, Abu-Raddad LJ. The epidemiology of hepatitis C virus in the Fertile Crescent: systematic review and meta-analysis. PloS one. 2015;10(8):e0135281.

3. Mohamoud YA, Riome S, Abu-Raddad LJ. Epidemiology of hepatitis C virus in the Arabian Gulf countries: Systematic review and meta-analysis of prevalence. International Journal of Infectious Diseases. 2016;46:116–25.

4. Chaabna K, Kouyoumjian SP, Abu-Raddad LJ. Hepatitis C Virus Epidemiology in Djibouti, Somalia, Sudan, and Yemen: Systematic Review and Meta-Analysis. PLoS One. 2016;11(2):e0149966.

5. Azzam A, Khaled H, Hussein SM, Gendy KR, Hassan FE. Prevalence and clinical implications of occult hepatitis C infection: a meta-analysis of studies conducted in Egypt. The Egyptian Journal of Internal Medicine. 2025;37(1):1–12.

6. Abdel‐Gawad M, Abd‐elsalam S, Abdel‐Gawad I, Tag‐Adeen M, El‐Sayed M, Abdel‐Malek D. Seroprevalence of hepatitis C virus infection in children: A systematic review and meta‐analysis. Liver International. 2022;42(6):1241–9.

7. Ghaderi-Zefrehi H, Sharafi H, Sadeghi F, Gholami-Fesharaki M, Farasat A, Jahanpeyma F, et al. Seroprevalence of Hepatitis C virus among blood donors in middle eastern countries: A systematic review and meta-analysis. Iran Red Crescent Med J. 2017;19:e58045.

8. Kouyoumjian SP, Chemaitelly H, Abu-Raddad LJ. Characterizing hepatitis C virus epidemiology in Egypt: systematic reviews, meta-analyses, and meta-regressions. Scientific reports. 2018;8(1):1661.

9. Obeid D, Alsuwairi F, Alnemari R, Al-Qahtani A, Kurdi W, Alfareh M, et al. Sexually transmitted infections in the middle east and North Africa: comprehensive systematic review and meta-analysis. BMC infectious diseases. 2024;24(1):1229.

10. Kasraian L, Imanieh MH, Tabrizi R, Shahriarirad R, Erfani A, Hosseini S. Prevalence of hbv and hcv infections in iranian blood donors; an updated systematic review and meta-analysis. Middle East journal of digestive diseases. 2021;13(3):237.

11. Mirminachi B, Mohammadi Z, Merat S, Neishabouri A, Sharifi AH, Alavian SH, et al. Update on the prevalence of hepatitis c virus infection among iranian general population: A systematic review and meta-analysis. Hepatitis Monthly. 2017;17(2).

12. Bagheri Amiri F, Mostafavi E, Mirzazadeh A. HIV, HBV and HCV Coinfection Prevalence in Iran--A Systematic Review and Meta-Analysis. PLoS One. 2016;11(3):e0151946.

13. Khodabandehloo M, Roshani D, Sayehmiri K. Prevalence and trend of hepatitis C virus infection among blood donors in Iran: A systematic review and meta-analysis. J Res Med Sci. 2013;18(8):674–82.

14. Fadlalla FA, Mohamoud YA, Mumtaz GR, Abu-Raddad LJ. The epidemiology of hepatitis C virus in the Maghreb region: systematic review and meta-analyses. PloS one. 2015;10(3):e0121873.

15. Mahmud S, Al Kanaani Z, Abu-Raddad LJ. Characterization of the hepatitis C virus epidemic in Pakistan. BMC infectious diseases. 2019;19(1):809.

16. Al Kanaani Z, Mahmud S, Kouyoumjian SP, Abu-Raddad LJ. The epidemiology of hepatitis C virus in Pakistan: systematic review and meta-analyses. Royal Society open science. 2018;5(4):180257.

17. Ali Mude AS, Nageye YA, Bello KE. Prevalence of hepatitis C viral infection in Somalia: A systematic review and meta-analysis. Microbes and Infectious Diseases. 2025;6(2):515–30.

18. Hassan-Kadle MA, Osman MS, Ogurtsov PP. Epidemiology of viral hepatitis in Somalia: systematic review and meta-analysis study. World journal of gastroenterology. 2018;24(34):3927.

19. Hedayati-Moghaddam MR, Soltanian H, Ahmadi-Ghezeldasht S. Occult hepatitis C virus infection in the Middle East and Eastern Mediterranean countries: A systematic review and meta-analysis. World Journal of Hepatology. 2021;13(2):242.

20. Harfouche M, Chemaitelly H, Mahmud S, Chaabna K, Kouyoumjian S, Al Kanaani Z, et al. Epidemiology of hepatitis C virus among hemodialysis patients in the Middle East and North Africa: systematic syntheses, meta-analyses, and meta-regressions. Epidemiology & Infection. 2017;145(15):3243–63.

21. Ashkani-Esfahani S, Alavian SM, Salehi-Marzijarani M. Prevalence of hepatitis C virus infection among hemodialysis patients in the Middle-East: A systematic review and meta-analysis. World J Gastroenterol. 2017;23(1):151–66.

22. Alavian S-M, Tabatabaei S-V, Mahboobi N. Epidemiology and risk factors of HCV infection among hemodialysis patients in countries of the Eastern Mediterranean Regional Office of WHO (EMRO): a quantitative review of literature. Journal of Public Health. 2011;19(2):191–203.

23. Alavian S, Tabatabaei S, BAGHERI LK. Epidemiology of HCV infection among thalassemia patients in eastern Mediterranean countries: a quantitative review of literature. 2010.

24. Molaei A, Fesharaki MG. Epidemiology of Hepatitis C Virus in Iranian Thalassemic, Hemodialysis and Hemophiliac Patients: A Meta-Analysis Study. Journal of Biostatistics and Epidemiology. 2021.

25. Shamshirian A, Alizadeh-Navaei R, Pourfathollah AA, Alipoor R, Mohseni AR. Seroprevalence and geographical distribution of hepatitis C virus in Iranian patients with thalassemia: a systematic review and meta-analysis. Journal of Laboratory Medicine. 2019;43(2):45–55.

26. Behzadifar M, Gorji HA, Bragazzi NL. The prevalence of hepatitis C virus infection in thalassemia patients in Iran from 2000 to 2017: a systematic review and meta-analysis. Arch Virol. 2018;163(5):1131–40.

27. Ramezan Ghorbani N, Qorbani M, Djalalinia S, Kazemzadeh Atoofi M, Tajbakhsh R, Mansourian M, et al. Oncogenic Viral Infections Among Iranian Hemodialysis Patients: A Systematic Review. Int J Prev Med. 2019;10:216.

28. Liu Y-B, Xie J-Z, Zhong C-J, Liu K. Hepatitis C virus infection among hemodialysis patients in Asia: a meta-analysis. European Review for Medical & Pharmacological Sciences. 2014;18(21).

29. Alavian SM, Aalaei-Andabili SH. Lack of knowledge about hepatitis C infection rates among patients with inherited coagulation disorders in countries under the Eastern Mediterranean Region Office of WHO (EMRO): a meta-analysis. Hepatitis monthly. 2012;12(4):244.

30. ALAVIAN SM, Kabir A, Ahmadi AB, Lankarani KB, Shahbabaie MA, AHMADZAD‐ASL M. Hepatitis C infection in hemodialysis patients in Iran: a systematic review. Hemodialysis International. 2010;14(3):253–62.

31. Ashkani-Esfahani S, Alavian SM, Salehi-Marzijarani M. Prevalence of hepatitis C virus infection among hemodialysis patients in the Middle-East: A systematic review and meta-analysis. World journal of gastroenterology. 2017;23(1):151.

32. Waheed U, Saba N, Wazeer A, Ahmed S. A Systematic Review and Meta-Analysis on the Epidemiology of Hepatitis B and Hepatitis C Virus among Beta-Thalassemia Major Patients in Pakistan. J Lab Physicians. 2021;13(3):270–6.

33. Akhtar S, Nasir JA, Hinde A. The prevalence of hepatitis C virus infection in β-thalassemia patients in Pakistan: a systematic review and meta-analysis. BMC Public Health. 2020;20(1):587.

34. Akhtar S, Nasir JA, Usman M, Sarwar A, Majeed R, Billah B. The prevalence of hepatitis C virus in hemodialysis patients in Pakistan: A systematic review and meta-analysis. PloS one. 2020;15(5):e0232931.

35. Mahmud S, Chemaitelly H, Al Kanaani Z, Kouyoumjian SP, Abu‐Raddad LJ. Hepatitis C Virus Infection in Populations With Liver‐Related Diseases in the Middle East and North Africa. Hepatology Communications. 2020;4(4):577–87.

36. Hashiani AA, Sadeghi F, Ayubi E, Rezaeian S, Moradi Y, Mansori K, et al. Prevalence of HIV, hepatitis B and C virus co-infections among Iranian high-risk groups: a systematic review and meta-analysis. The Malaysian journal of medical sciences: MJMS. 2019;26(3):37.

37. Adeiza SS, Suleiman AS, Islam MA, Aminul IM, Mungadi HuU, Umar MHu, et al. A preregistered meta-meta-analysis on the global distribution of Hepatotropic Viruses. Problems of Virology. 2024;69(5):429–40.

38. Aghaei AM, Gholami J, Sangchooli A, Rostam-Abadi Y, Olamazadeh S, Ardeshir M, et al. Prevalence of injecting drug use and HIV, hepatitis B, and hepatitis C in people who inject drugs in the Eastern Mediterranean region: a systematic review and meta-analysis. The Lancet Global Health. 2023;11(8):e1225–e37.

39. Shayan SJ, Nazari R, Kiwanuka F. Prevalence of HIV and HCV among injecting drug users in three selected WHO-EMRO countries: a meta-analysis. Harm reduction journal. 2021;18(1):59.

40. Mahmud S, Mumtaz GR, Chemaitelly H, Al Kanaani Z, Kouyoumjian SP, Hermez JG, et al. The status of hepatitis C virus infection among people who inject drugs in the Middle East and North Africa. Addiction. 2020;115(7):1244–62.

41. Kouyoumjian S, Chemaitelly H, Abu-Raddad L. Characterizing hepatitis C virus epidemiology in Egypt: systematic reviews, meta-analyses, and meta-regressions. Sci Rep. 2018; 8 (1): 1661. Epub 2018/01/28.

42. Mostafavi E, Ebrahimi B, Doosti-Irani A, Mirzazadeh A. Prevalence of hepatitis B, hepatitis C, and tuberculosis among people living with HIV in Iran: a systematic review and meta-analysis. BMC Infect Dis. 2024;24(1):777.

43. Hajizadeh M, Binabaj MM, Asadi A, Abdi M, Shakiba A, Beig M, et al. Prevalence of HCV among patients with HIV in Iran: A systematic review and meta-analysis. Vacunas (English Edition). 2024;25(1):128–39.

44. Nasiri N, Kostoulas P, Roshanfekr P, Kheirkhah Vakilabad AA, Khezri M, Mirzaei H, et al. Prevalence of HIV, hepatitis B virus, hepatitis C virus, drug use, and sexual behaviors among street children in Iran: A systematic review and meta-analysis. Health Sci Rep. 2023;6(11):e1674.

45. Mehmandoost S, Khezri M, Mousavian G, Tavakoli F, Mehrabi F, Sharifi H, et al. Prevalence of HIV, hepatitis B virus, and hepatitis C virus among incarcerated people in Iran: a systematic review and meta-analysis. Public health. 2022;203:75–82.

46. Rajabi A, Sharafi H, Alavian SM. Harm reduction program and hepatitis C prevalence in people who inject drugs (PWID) in Iran: an updated systematic review and cumulative meta-analysis. Harm Reduction Journal. 2021;18(1):12.

47. Karamouzian M, Nasirian M, Ghaffari Hoseini S, Mirzazadeh A. HIV and Other Sexually Transmitted Infections Among Female Sex Workers in Iran: A Systematic Review and Meta-Analysis. Arch Sex Behav. 2020;49(6):1923–37.

48. Najimi A, Gholami-Fesharaki M, Rowzati M. Prevalence of hepatitis C virus in Iranian prisoners: an updated systematic review and multilevel meta-analysis study. Hepatitis Monthly. 2020;20(5).

49. Najimi-Varzaneh A, Gholami-Fesharaki M. Prevalence and Distribution of Hepatitis C Virus in Iranian Drug User: Systematic Review and Meta-Analysis Study. Journal of Biostatistics and Epidemiology. 2020;6(1):72–80.

50. Behzadifar M, Gorji HA, Rezapour A, Bragazzi NL. Prevalence of hepatitis C virus among street children in Iran. Infectious diseases of Poverty. 2018;7(05):77–82.

51. Behzadifar M, Gorji HA, Rezapour A, Bragazzi NL. Prevalence of hepatitis C virus infection among prisoners in Iran: a systematic review and meta-analysis. Harm Reduct J. 2018;15(1):24.

52. MOHAMMADI M, MIRZAEI M, MAHMOUDI KHA, ZAHEDNEZHAD H, VAISI RAA, JALALI A, et al. PREVALENCE OF HEPATITIS C IN IRANIAN PRISONERS (2001-2016): A SYSTEMATICREVIEW AND META-ANALYSIS. 2018.

53. Nematollahi S, Ayubi E, Almasi-Hashiani A, Mansori K, Moradi Y, Veisani Y, et al. Prevalence of hepatitis C virus infection among high-risk groups in Iran: a systematic review and meta-analysis. Public Health. 2018;161:90–8.

54. Malekinejad M, Navadeh S, Lotfizadeh A, Rahimi-Movaghar A, Amin-Esmaeili M, Noroozi A. High hepatitis C virus prevalence among drug users in Iran: systematic review and meta-analysis of epidemiological evidence (2001–2012). International Journal of Infectious Diseases. 2015;40:116–30.

55. Jin F, Dore GJ, Matthews G, Luhmann N, Macdonald V, Bajis S, et al. Prevalence and incidence of hepatitis C virus infection in men who have sex with men: a systematic review and meta-analysis. The lancet Gastroenterology & hepatology. 2021;6(1):39–56.

1. Based on the original AMSTAR tool (A Measurement Tool to Assess Systematic Reviews) [↑](#footnote-ref-1)
2. Includes Saudi Arabia, United Arab Emirates, Kuwait, Qatar, Oman, and Bahrain [↑](#footnote-ref-2)
3. Includes Saudi Arabia, United Arab Emirates, Kuwait, Qatar, Oman, and Bahrain [↑](#footnote-ref-3)
