# Supplementary figures and images for: Hepatitis C distribution across diverse population groups in the Eastern Mediterranean Region: An umbrella review
Source: PLoS One. 2026 Apr 21;21(4):e0346782. doi: 10.1371/journal.pone.0346782 (PMC13098937; doi:10.1371/journal.pone.0346782)

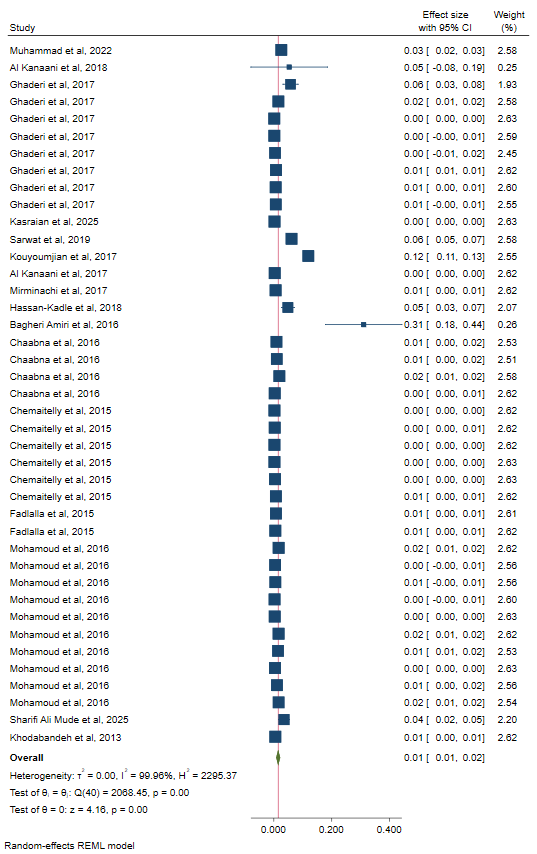

Supplement: S1 Fig — (TIF) [file pone.0346782.s004.tif]

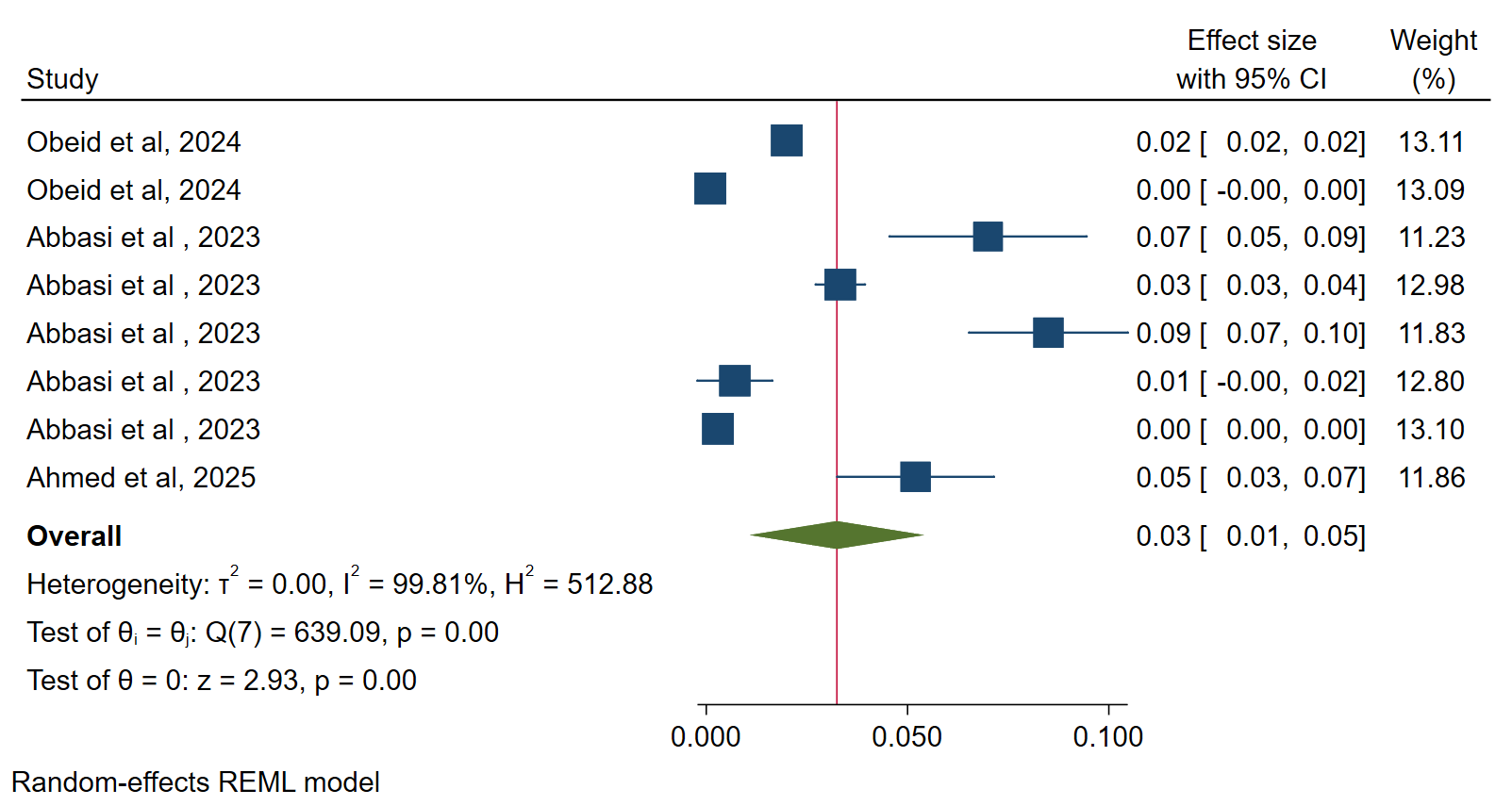

Supplement: S2 Fig — (TIF) [file pone.0346782.s005.tif]

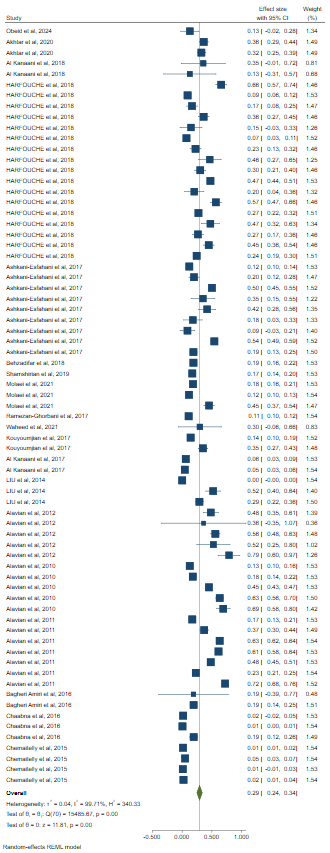

Supplement: S3 Fig — (TIF) [file pone.0346782.s006.tif]

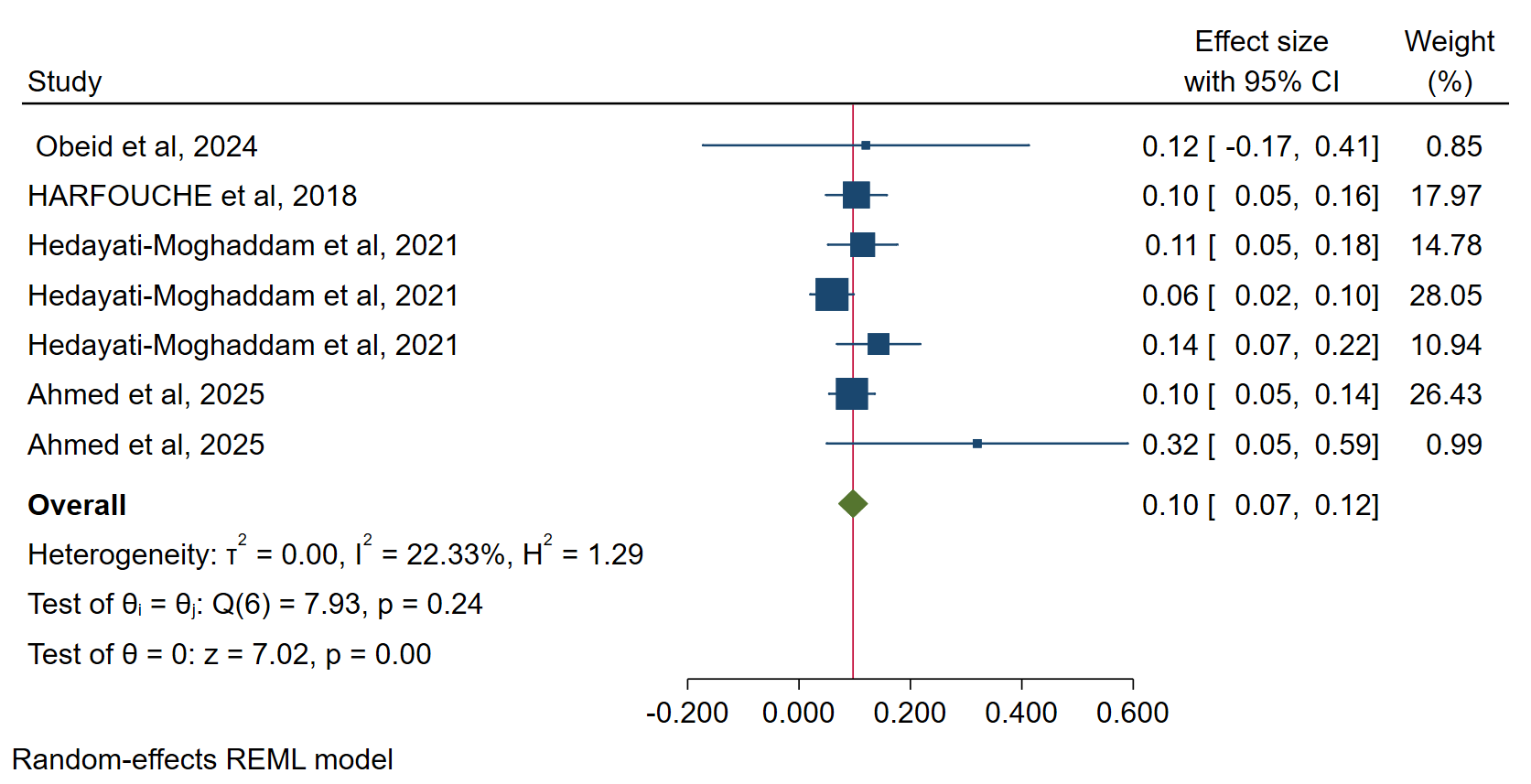

Supplement: S4 Fig — (TIF) [file pone.0346782.s007.tif]

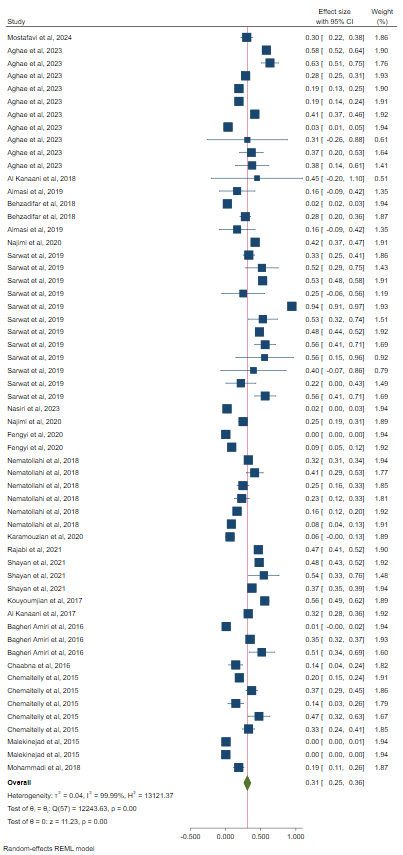

Supplement: S5 Fig — (TIF) [file pone.0346782.s008.tif]

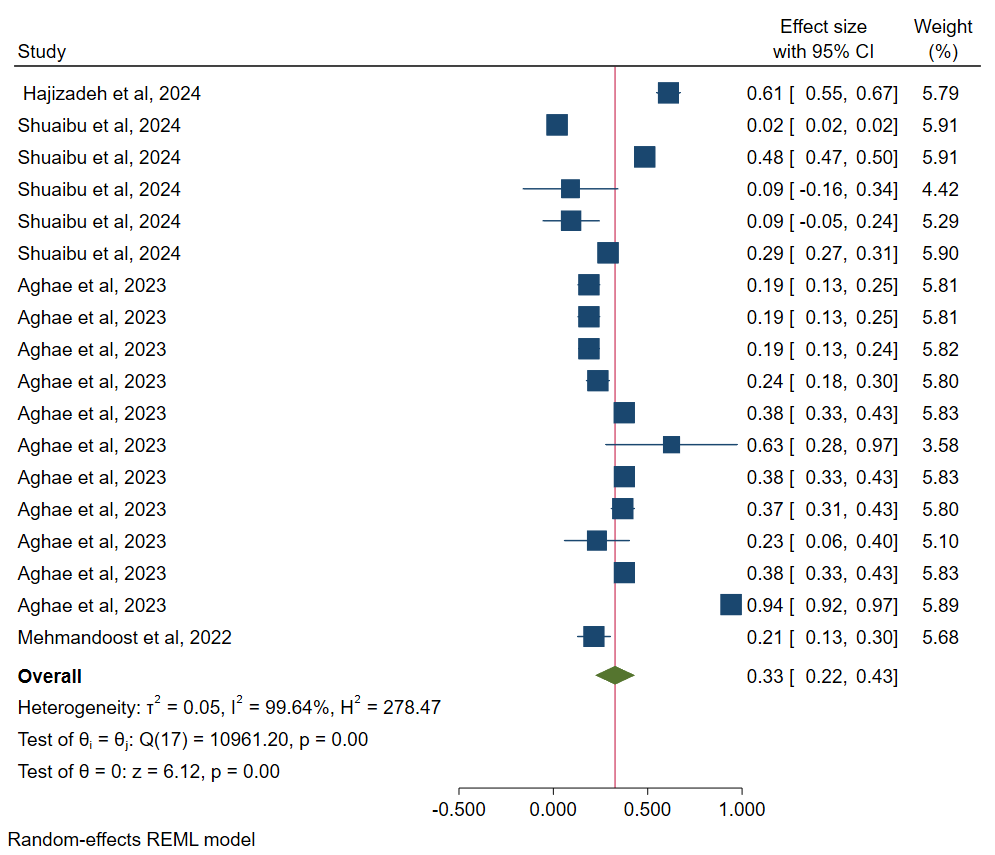

Supplement: S6 Fig — (TIF) [file pone.0346782.s009.tif]
