# Supplementary material for: Hepatitis C distribution across diverse population groups in the Eastern Mediterranean Region: An umbrella review
Source: PLoS One. 2026 Apr 21;21(4):e0346782. doi: 10.1371/journal.pone.0346782 (PMC13098937; doi:10.1371/journal.pone.0346782)
Supplement: Attachment — Submitted filename: Rebuttal letter.docx [file pone.0346782.s010.docx]

Dear Editor,

We sincerely thank you for your time and for the constructive feedback provided on our previous submission entitled “*Hepatitis C Distribution Across Diverse Population Groups in the Eastern Mediterranean Region: An Umbrella Review”* (Manuscript ID: PONE-D-25-67259).

Below, we provide a point-by-point response to the editorial comment:

**Valuable Comment:**

In the case of your submission, we note that language restrictions are imposed on the literature searches without adequate justification. There is increasing recognition that exclusion of studies published in other languages can increase the risk of bias in systematic reviews. We feel that any restrictions should be justified based on scientific reasons rather than resource constraints. As a result, we have concerns about the comprehensiveness of the review and the validity of the conclusions.

**Response:**

We carefully considered the editorial comment regarding the restriction of the literature search to English-language publications. In response, we have revised our search strategy to remove all language restrictions and improve our search. As a result of this expanded search, one additional eligible meta-analysis was identified and included in the umbrella review. The Methods, Results, tables, and figures have been updated accordingly to fully incorporate this additional evidence. The revised search strategy and corresponding changes are described in detail in the manuscript (Methods section and in the Supplementary Materials).

We hope that these revisions have strengthened the methodology and completeness of the review and have fully addressed the editorial concern.

We appreciate the opportunity to resubmit our revised manuscript for reconsideration in PLOS ONE.

Sincerely,

Hamid Sharifi

HIV/STI Surveillance Research Center, and WHO Collaborating Center for HIV Surveillance, Institute for Futures Studies in Health, Kerman University of Medical Sciences, Kerman, Iran

E-mail: [sharifihami@gmail.com](mailto:sharifihami@gmail.com)
